# Case Report: Next-Generation Sequencing-Based Detection in A Patient with Three Synchronous Primary Tumors

**DOI:** 10.3389/fonc.2022.910264

**Published:** 2022-07-15

**Authors:** Tianqi Wu, Jian Wan, Kai Xia, Muqing Yang, Lijin Feng, Lu Yin, Chunqiu Chen

**Affiliations:** ^1^ Center for Difficult and Complicated Abdominal Surgery, Shanghai Tenth People’s Hospital, Tongji University School of Medicine, Shanghai, China; ^2^ Department of Pathology, Shanghai Tenth People’s Hospital, Tongji University School of Medicine, Shanghai, China

**Keywords:** MPT, BRCA2, GIST, BC, NGS

## Abstract

Clinically rare, multiple primary tumors are a growth or development of two or more neoplasms in the same individual. A 57-year-old woman with two primary cancers, namely, breast and gastric cancers, and a gastrointestinal stromal tumor was admitted. Next-generation sequencing (NGS) of the three tumors and blood was performed to determine their clonal origin and identify genetic cancer susceptibility. NGS identified that germline genetic variants potentially correlated with an individual risk of developing multiple cancers and that additional mutations are required to drive the formation of different tumors.

## Introduction

Multiple primary tumors (MPTs) are characterized by two or more separate synchronous or metachronous neoplasms of different primary disease sites and histology in the same individual (1). Next-generation sequencing (NGS) accurately detects hereditary germline mutations and acquired somatic mutations, providing personalized prevention programs and follow-ups for patients with a high predisposition.

## Case description

**Figure 1:**
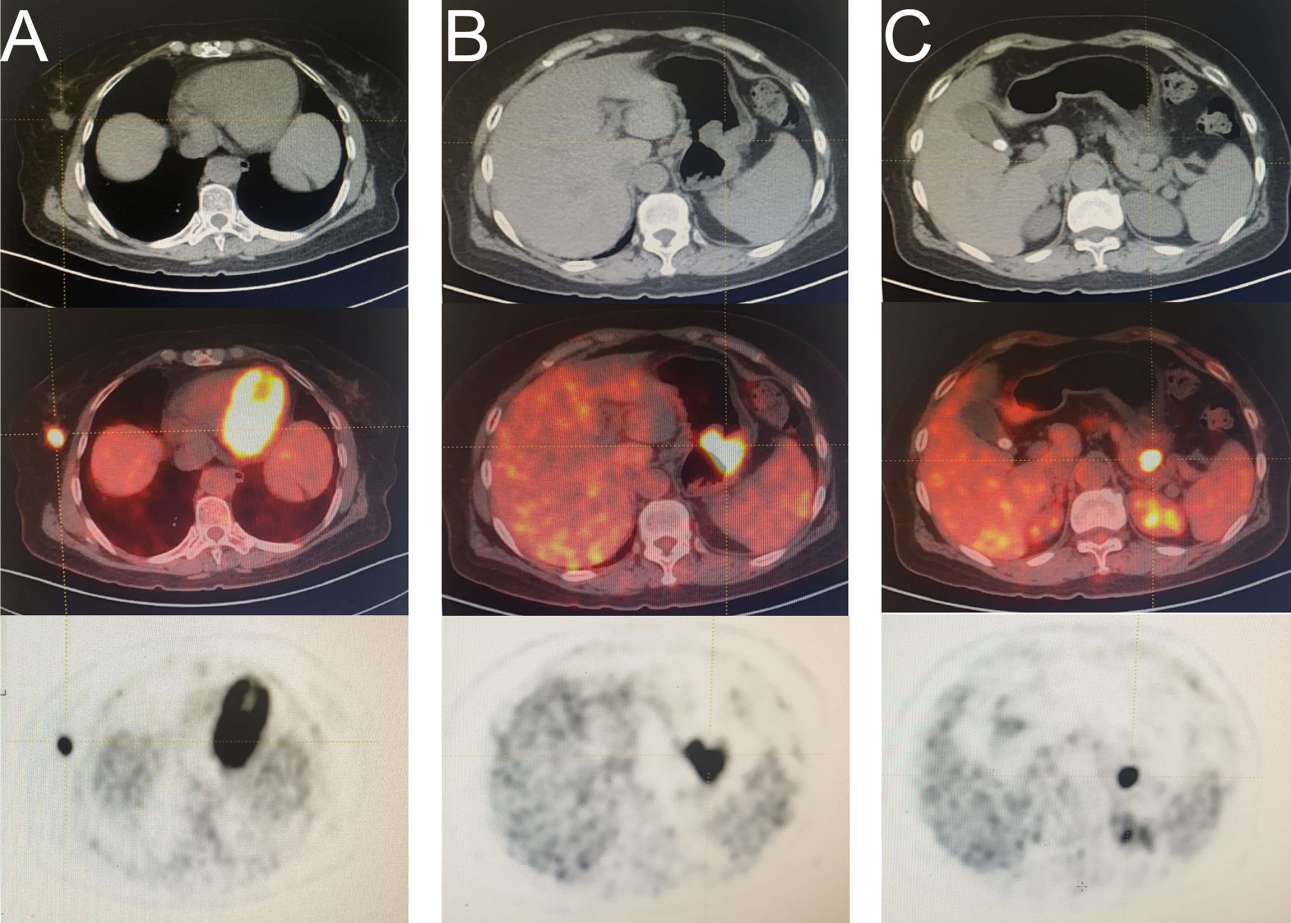
A F18-fluorodeoxyglucose (FDG) positron emission tomography computed tomography (PET-CT) scan was conducted. **(A)** mass in right breast **(B)** mass in the greater curvature of the stomach **(C)** mass in the space between the stomach and pancreas.

A 57-year-old woman with abdominal pain for a month was admitted to our hospital on March 18, 2021. A mass was found in the greater curvature using gastric endoscopy, and adenocarcinoma was identified *via* pathological examination. In addition, a nodule was found in the right mammary gland, pathologically confirming an infiltrating ductal carcinoma. A F18-fluorodeoxyglucose positron emission tomography-computed tomography scan was conducted (**Figure 1**). Laparoscopic total gastrectomy (Roux-en-Y) was performed on March 25, 2021. After incision of the specimen, two tumors were found in the stomach body, one in the cavity and one outside the wall (**Supplementary Figure 1**). After more than a month of recovery, right breast cancer-modified radical mastectomy was performed on May 6, 2021, and five sentinel lymph nodes were diagnosed as negative upon intraoperative frozen sections. Pathological findings showed that the patient had primary breast and gastric cancers with gastrointestinal stromal tumor (GIST) (**Supplementary Table 1**).

NGS of DNA obtained from peripheral blood and three tumors was performed to identify possible germline mutations and acquired somatic mutations. Details of the NGS and data analysis methods are provided in the supplementary material. Seven possible pathogenic germline mutations were detected in the blood, namely, ERCC6, SLCO1B1, ALDH2, BRCA2, CNGA1, PRSS12, and PEX1 (**Supplementary Table 2**). In addition, 104, 42, and 166 somatic mutations were detected in breast cancer, gastric cancer, and GIST, respectively (**Supplementary Tables 3A–C**). Interestingly, three gene mutations, namely, RIMS3, LOC283710, and ABCC6, were present in all three tumors (**Table 1**). GIST and gastric cancer carried KIT (COSM1169) and TP53 (COSM10731) hotspot mutations, respectively, which were reported in the Catalogue of Somatic Mutations in Cancer (COSMIC) database (http://cancer.sanger.ac.uk/cosmic). However, breast cancer did not show any COSMIC mutations associated with carcinogenesis (**Table 2**). Although BRCA2 germline mutation (p.Y1894*) was detected in blood and all three tumors, a new BRCA2 somatic mutation (p.S744X) was detected in gastric cancer. Loss of heterozygosity (LOH) event was identified in the pathogenic BRCA2 germline mutation in the breast cancer sample alone, indicating the driving role of this mutation in breast cancer. Although the LOH of BRCA2 was absent in the gastric cancer sample, somatic mutation of BRCA2 may contribute as a second-hit event of BRCA2.

**Table 1:** Summary of the similar somatic mutations existing in each tumor analyzed by NGS.

Ref. gene	ExonicFunc.refGene	#Chr	Start	End	Ref	Alt	avsnp147
RIMS3	nonsynonymous SNV	1	41094951	41094951	1	1	rs764627942
LOC283710	frameshift deletion	15	31521506	31521506	15	15	rs34406656
ABCC6	nonsynonymous SNV	16	16281007	16281007	16	16	rs12931472

**Table 2:** Summary of their specific driven mutations existing in each tumor analyzed by NGS.

Location	Ref. gene	Transcript	Exon	cHGVS	pHGVS
GIST	KIT	NM_000222.2	exon11	c.1652_1666del	p.P551_V555del
Gastric cancer	BRCA2	NM_000059.3	exon11	c.2231C>A	p.S744*
	TP53	NM_000546.5	exon7	c.707A>G	p.Y236C
Breast cancer	No typical driven mutation was found				

Thus far, the patient underwent four cycles of TC regimen (cyclophosphamide 600 mg/m^2^, docetaxel 75 mg/m^2^) chemotherapy-targeted breast cancer. The final chemotherapy was performed on August 25, 2021. No recurrence was observed during the chemotherapy and after a 9-month follow-up period post chemotherapy (**Supplementary Figure 2**).

## Discussion

With the advancement of early-stage cancer detection, the incidence of MPTs is increasing. Factors contributing to MPTs include congenital factors, such as genetic susceptibility, familial cancer syndromes, and immune deficiency; acquired factors, such as environment, lifestyle exposures, and infection; and interaction among all these factors (2, 3). Here, we described the first case of a patient with synchronous development of breast cancer, gastric cancer, and GIST.

NGS of blood and three tumors was performed to determine a clonal relationship between three tumors. NGS of the blood showed that the patient carried seven frequent pathogenic germline mutations, namely, ERCC6, SLCO1B1, ALDH2, BRCA2, CNGA1, PRSS12, and PEX1. Of these, BRCA2 stop gain mutation p.Y1894* was the only possible pathogenic variant in this case. ERCC6, a gene involved in preferential repair, is generally closely related to Cockayne’s syndrome (4). However, recent reports show that ERCC6 leads to increased predisposition to breast and gastric cancers (4–6). ALDH2 rs671 G>A polymorphism reportedly increases gastric cancer susceptibility in alcohol consumers (7–9), but the patient in this case study had no history of alcohol abuse. Mutations in the remaining four genes, namely, SLCO1B1, CNGA1, PRSS12, and PEX1, have associations with autosomal diseases, such as autosomal recessive retinitis pigmentosa and non-syndromic intellectual disability (10, 11). Hence, they are not associated with tumors. The tumor-susceptibility gene BRCA2 with high penetrance is primarily concerned with the homologous recombination mechanism of DNA repair, and its variants are the leading causes of familial and early-onset breast cancer (12). Recently, an association between BRCA2 and gastric cancer has been reported (13). However, reports on the relationship between BRCA2 mutation and GIST are scarce.

Although tumor development in multiple locations attributing to a common gene mutation has been proposed, it is not proven yet. A study reported that the association among tumors is due to specific driver mutations in different tumors or a coincidence (14). In the present study, we explore whether additional somatic mutations exist to drive the formation of various tumors. NGS analysis of the three tumors showed that three genes, namely, RIMS3, LOC283710, and ABCC6, with the same somatic mutation, were present in each tumor of the same individual. Previous studies showed no relationship between the three genes and the three tumors, suggesting that these mutations may be random. Furthermore, TP53 and KIT driver mutations were found in gastric cancer and GIST, respectively, suggesting that gastric cancer and GIST possibly emerge from somatic mutations of TP53 and KIT, respectively. However, the pathogenesis of BRCA2 p.Y1894* germline mutation should not be neglected. Considering the LOH event of BRCA2 in breast cancer and BRCA2 p.S744X somatic mutation in gastric cancer, BRCA2 p.Y1894* mutation is prone to a second-hit event of BRCA2 in them. Two copies of BRCA2 get inactivated subsequently, forming a driving factor for the inactivation of the tumor-suppressor gene and promoting neoplasia. It is speculated that TP53 may not be the only driving mutation in gastric cancer, and the second-hit event caused by BRCA2 mutation may be involved, confirming the relationship between BRCA2 and gastric cancer. Therefore, the BRCA2 pathogenic variant led to the systemic predisposition to malignancy in the present study, which is crucial for precision therapy and prevention of the patient and her family members.

Mutations in RIMS3, LOC283710, and ABCC6 genes do not cause tumor formation, suggesting that they might be passenger mutations. The current observation is that a typical tumor contains two to eight driver gene mutations, with a major effect on tumor development; the remaining are passenger mutations with no selective growth advantage (15). Nevertheless, a recent study demonstrate that a third group of medium-impact putative passengers exist in addition to the dichotomy of high- and low-impact variants, possibly exerting a weak driving force on tumorigenesis. With tumor development, the cumulative effect of these genes will be prominent even in the later stage of tumor, exceeding the influence of driving mutation to play a dominant role (15). However, further studies are required to elucidate whether RIMS3, LOC283710, and ABCC6 genes are medium-impact putative passengers involved in the molecular pathways of carcinogenesis, leading to gastric cancer, breast cancer, or GIST.

The present study reported the case of a patient with MPT and performed NGS to identify the genetic variants leading to cancer susceptibility and specific carcinogenesis. NGS showed the crucial effect of BRCA2 p.Y1894* mutation on tumorigenesis. This indicates that if the patient exhibits tumor recurrence and metastasis in future follow-up, targeted BRCA2 therapy may be used. This confirms the benefit of NGS in defining the clonal feature and discovering novel potential molecular targets for subsequent treatment.

## Data Availability Statement

The original contributions presented in the study are included in the article/**Supplementary Materials**. Further inquiries can be directed to the corresponding authors.

## Ethics Statement

The studies involving human participants were reviewed and approved by Ethics Committee of Shanghai Tenth People’s Hospital. The patients/participants provided their written informed consent to participate in this study. Written informed consent was obtained from the individual(s) for the publication of any potentially identifiable images or data included in this article.

## Author Contributions

TW: writing manuscript and collect samples. JW: manuscript review and communication with patient. KX and MY: manuscript review, resident in charge of the patient during treatment. LF: provided pathology report. CC: supervised the writing of the paper and patient treatment. LY: provide financial support to NGS. All authors contributed to the article and approved the submitted version.

## Conflict of Interest

The authors declare that the research was conducted in the absence of any commercial or financial relationships that could be construed as a potential conflict of interest.

## Publisher’s Note

All claims expressed in this article are solely those of the authors and do not necessarily represent those of their affiliated organizations, or those of the publisher, the editors and the reviewers. Any product that may be evaluated in this article, or claim that may be made by its manufacturer, is not guaranteed or endorsed by the publisher.
