# Supplementary material for: Case Report: Next-Generation Sequencing-Based Detection in A Patient with Three Synchronous Primary Tumors
Source: Front Oncol. 2022 Jul 15;12:910264. doi: 10.3389/fonc.2022.910264 (PMC9334672; doi:10.3389/fonc.2022.910264)
Supplement: Supplementary file 2 [file DataSheet_2.docx]

# NGS and Data analysis

Genomic DNA was typically extracted from peripheral blood samples and three tumor tissues using the HiPure FFPE DNA kit (Magen, Guangzhou, China) and the DNA was quantified and concentrated according to the manufacturer’s instructions. The amount of DNA required for detection was 50-150ng. Next, the DNA was interrupted into fragments with an average size of ~ 200 bp. Quality control was performed prior to sequencing.

The library was sequenced by hybridization with NimbleGen SeqCap EZ Exome Library. The capture beads were used to pull down the complex of capture oligonucleotides and genomic DNA fragments. Unbound fragments were eluted. The enriched fragment library was amplified by ligation-mediated PCR (LM-PCR). The average fragment size was about 320bp. The amplified DNA library concentration should be greater than 1ng/ul.

The original data (fastq.gz) file was compared to the human reference genome GRCh37 by BWA MEM (0.7.16a-r1181); The compared SAM files were sorted and mark duplicated by Sambamba (0.6.6) algorithm. The removed bam files were identified SNV/INDEL using freebayes (v 1.3.2) algorithm. Mutation QUAL>=1, covering depth > 10, and the mutations in the target area were reserved for further analysis and annotation. The SnpEff (4.3t) algorithm was used for mutation function annotation.

VarDict (v1.8.2) was used for somatic mutation, calling the pair analysis mode were used which utilized control and tumor bam to call and assign the somatic status of each variant. peripheral blood sample was used as control for somatic mutation calling. Variant labeled as “strongsomatic“ were used as somatic variants in further analysis. SNV/INDEL of somatic cells was identified. SnpEff(4.3t) algorithm was used for mutation function annotation.

LOH（loss of heterozygosity, loh）analysis were performed with Facets algorithm. For the BRCA2 p.Y1894* mutation, we observed loh event in the breast cancer sample but not in the other two tissues.
