# Supplementary figures and images for: Case Report: Next-Generation Sequencing-Based Detection in A Patient with Three Synchronous Primary Tumors
Source: Front Oncol. 2022 Jul 15;12:910264. doi: 10.3389/fonc.2022.910264 (PMC9334672; doi:10.3389/fonc.2022.910264)

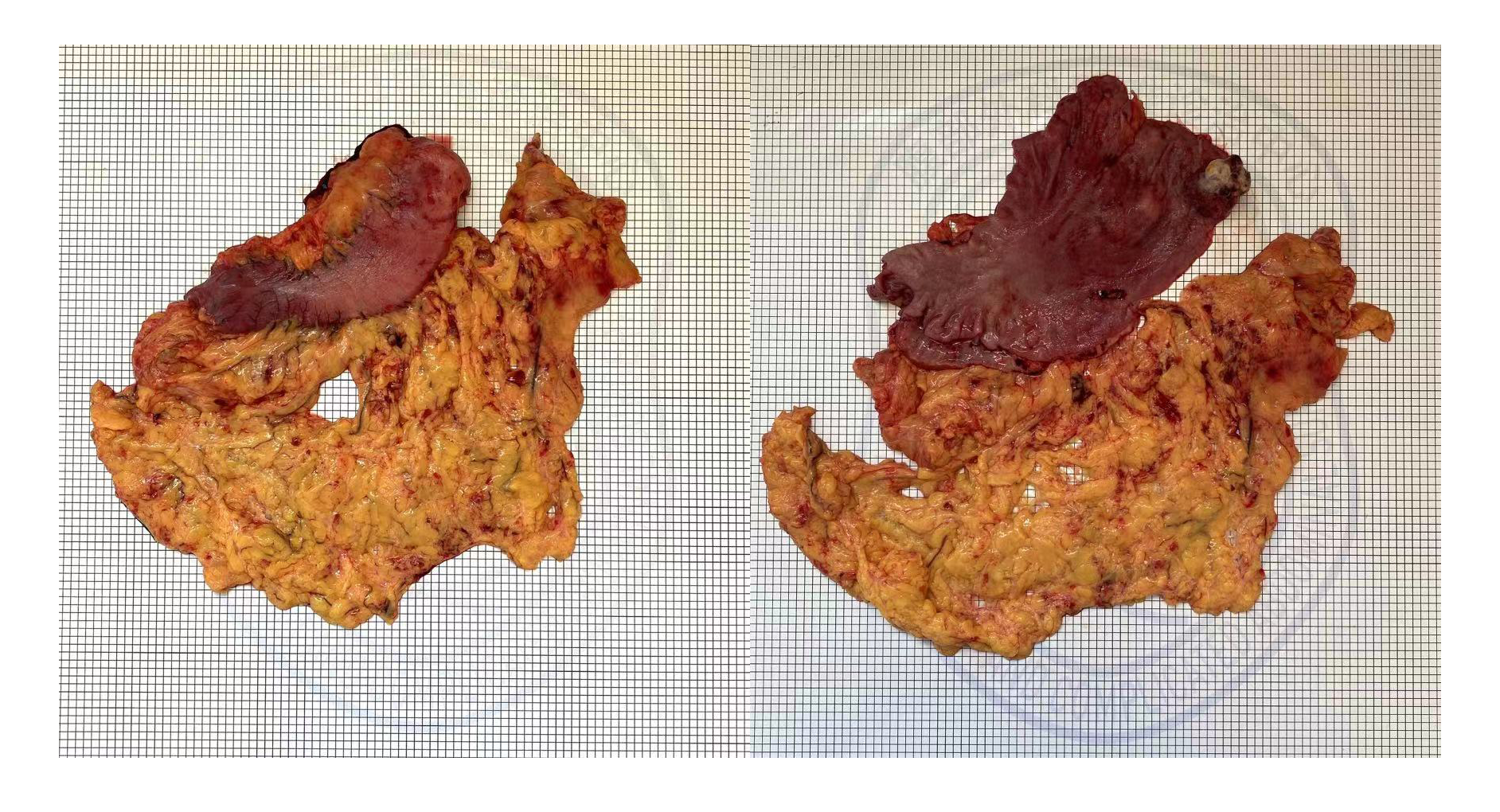

Supplement: Supplementary file 3 [file Image_1.tif]

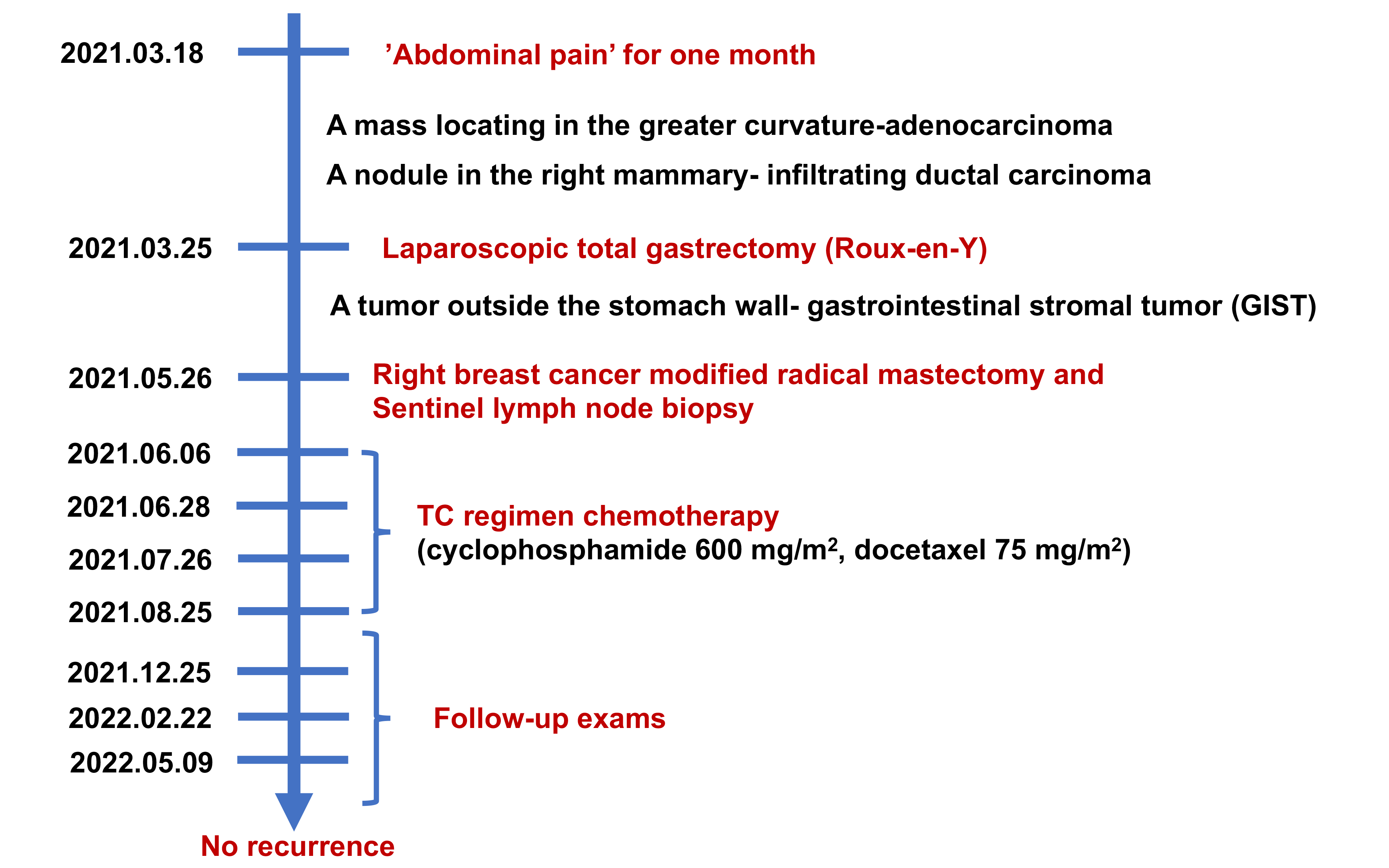

Supplement: Supplementary file 4 [file Image_2.tif]
